# Effectiveness of Guided Self-Help Versus Internet-Delivered or Face-to-Face Cognitive Behavioral Therapy for Depression and Anxiety: a Randomized Controlled Non-Inferiority Trial of the Finnish First-Line Therapies –Initiative (FLT-Step)

**DOI:** 10.1192/j.eurpsy.2025.687

**Published:** 2025-08-26

**Authors:** E.-E. Helminen, K. Mikkonen, S. I. Saarni, K. Mattila, T. Rosenström, E. Isometsä, S. E. Saarni

**Affiliations:** 1 Psychiatry, HUS Helsinki University Hospital; 2 University of Helsinki, Helsinki, Finland

## Abstract

**Introduction:**

Depression and anxiety are among the most prevalent mental health conditions, particularly in Western countries. Access to effective treatments, such as cognitive behavioral therapy (CBT), in publicly funded primary care settings is often insufficient.

**Objectives:**

This study aims to evaluate, within a non-inferiority framework, the effectiveness and cost-effectiveness of three treatment modalities for depression and anxiety within the Finnish public healthcare system: 1) a stepped care model, which involves sequential guided self-help (GSH) followed by face-to-face CBT for non-responders; 2) internet-delivered CBT (iCBT); and 3) face-to-face CBT.

**Methods:**

Our objective is to recruit 948 adults (16+ yrs) exhibiting symptoms of depression (scoring ≥10 on the Patient Health Questionnaire, PHQ-9) and 948 adults exhibiting symptoms of anxiety (scoring ≥10 on the Generalized Anxiety Disorder scale, GAD-7). These participants should be suitable for step 1 or step 2 treatments, such as GSH, iCBT or CBT, within the Finnish public healthcare system. Individuals currently receiving psychological treatment, those with severe suicidal ideation, cognitive impairment, or those with substance abuse issues will be excluded from the study. Participants will be randomly assigned to one of three groups: GSH, iCBT, or face-to-face CBT. Those who do not respond adequately to GSH will be stepped up for further treatment with face-to-face CBT. Participants will complete symptom measures, such as PHQ-9, GAD-7, measures of function, work ability and social support at various stages over the course of the study to track changes in their mental health. The trial and follow-up period will span 5 years. In addition to data collected from the study participants, the study incorporates direct and indirect healthcare, social care, employment, and societal cost data from Finnish national registries. These registries will also be utilized to create population-matched controls for the study participants. The study will be conducted within several wellbeing service counties in Southern Finland and Western Finland. It is part of the Finnish First-Line Therapies –initiative in Finland’s public healthcare system.

**Results:**

Recruitment of study participants began in autumn 2024 and is expected to take approximately one year. Initial results are anticipated by 2026. The study protocol has been registered in the ISRCTN registry prior to the commencement of participant recruitment. The study is primarily funded by grants from the Ministry of Social Affairs and Health in Finland (VN/29619/2023 and VN/29613/2023).

**Conclusions:**

The study aims to improve access to effective, evidence-based treatments for depression and anxiety within the public healthcare system.

**Disclosure of Interest:**

None Declared

